# Response to the letter from Dr J. Wang

**DOI:** 10.1017/S0950268820001375

**Published:** 2020-06-23

**Authors:** Z. Yuan, J. Jiang, A. Abdullah, H. Liang, L. Ye

**Affiliations:** 1 Guangxi Key Laboratory of AIDS Prevention and Treatment, School of Public Health, Guangxi Medical University, Nanning, Guangxi, China; 2 Boston University School of Medicine, Boston Medical Center, Boston, Massachusetts 02118, USA

Dear Editor,

Thank you for the opportunity to discuss the concerns raised by Dr J. Wang in relation to our publication. We appreciate the critiques which made some helpful comments on our study.

We disagree with the authors' claim that the mortality rates in our study were incredibly high. The mortality rates at the 10-day and 20-day periods were 5.2% and 9.2%, respectively [1]; this can be compared with the overall mortality rate of hospitalised HIV/AIDS patients in the training cohort of 9.02% [1]. These rates were percentages, not person-years or person-days. Regarding the data in Reference 3 of our paper, we stated a mortality rate of 29.36%, not 29.36 per 100 person-years. (From the original data, there were 74 deaths out of 252 records of HIV-infected patients admitted to the hospital during 2015 [2]). Hence, compared with the mortality rate in Reference 3 of our paper, the mortality rate in our study was much lower. Furthermore, HIV/AIDS patients in different regions of China are exposed to different regional opportunistic infections and the mortality rates for hospitalised HIV/AIDS patients from the different and various complications ranged from 0.7 to 33.7% [3]. Therefore, the mortality data in our study are consistent with those from across China.

Regarding our selection of the latest admission, we agree that only selecting data from the latest admission rather than multiple admissions could lead to an overestimation of mortality. We actually considered this issue during the data inclusion, which is not uncommon in predictive model studies. So far, many studies in the field have not described this process and there is no standard method to address this problem. We selected the latest admission for several reasons: (1) including the data from multiple admissions contains much repetitive information and may lead to greater bias; (2) some inpatients with multiple admissions had a very long span of up to 2–3 years between different admissions and we considered the information available in the latest admission to be more accurate; (3) due to the fact that the death can only appear in the latest hospital record, selection of the latest hospital record can also appropriately increase the power of statistical testing of the model, which could also facilitate more accurate detection of risk factors and timely treatment for the hospitalised HIV/AIDS patients. Furthermore, in our study, if multiple admissions are taken into consideration, the overall mortality rate of hospitalised HIV/AIDS patients was 8.9% (data not shown in the paper). Compared with the mortality rate of 9.2% based on latest admission, the difference (and the bias, if any) is relatively small and acceptable. Nevertheless, the issue of multiple admissions is indeed worth further study in the future.

In response to the discharge issue, the aim of our study was to predict the survival of hospitalised HIV/AIDS patients; therefore, the targeted event of the study was a death that occurred in the hospital. The death of patients after discharge was not the purpose of this study. Hospitalised HIV/AIDS patients in this study have two outcomes: death or discharge (the discharge was considered as the censored data), which is suitable for the Cox model. The Cox model, the most widely used survival regression model, can handle censored data [4]. Nevertheless, we agree that the censoring would cause a certain degree of bias, which we accept as inevitable.

In addition, we chose 10- and 20-day survival outcomes, based on the facts that the average time of hospitalisation was 18.2 days and the median time 15 days, which reflected the situation in the real-world settings. Although there may be some biases, we believe that our research is sound in principle. Furthermore, another main aim of this study was to develop a novel method based on nomogram to predict the survival of hospitalised HIV/AIDS patients, which is still scarce in the HIV/AIDS field. We hope that our study could ignite the inspiration of researchers in the field.

We would like to thank Dr Wang for his critique, which has provided us with further insights into model building. These concerns should be considered critically in future studies.
